# Similarity Ratio Analysis for Early Stage Fault Detection with Optical Emission Spectrometer in Plasma Etching Process

**DOI:** 10.1371/journal.pone.0095679

**Published:** 2014-04-22

**Authors:** Jie Yang, Conor McArdle, Stephen Daniels

**Affiliations:** Energy and Design Lab, School of Electronic Engineering, Dublin City University, Dublin, Ireland; University Paul Sabatier, France

## Abstract

A Similarity Ratio Analysis (SRA) method is proposed for early-stage Fault Detection (FD) in plasma etching processes using real-time Optical Emission Spectrometer (OES) data as input. The SRA method can help to realise a highly precise control system by detecting abnormal etch-rate faults in real-time during an etching process. The method processes spectrum scans at successive time points and uses a windowing mechanism over the time series to alleviate problems with timing uncertainties due to process shift from one process run to another. A SRA library is first built to capture features of a healthy etching process. By comparing with the SRA library, a Similarity Ratio (SR) statistic is then calculated for each spectrum scan as the monitored process progresses. A fault detection mechanism, named 3-Warning-1-Alarm (3W1A), takes the SR values as inputs and triggers a system alarm when certain conditions are satisfied. This design reduces the chance of false alarm, and provides a reliable fault reporting service. The SRA method is demonstrated on a real semiconductor manufacturing dataset. The effectiveness of SRA-based fault detection is evaluated using a time-series SR test and also using a post-process SR test. The time-series SR provides an early-stage fault detection service, so less energy and materials will be wasted by faulty processing. The post-process SR provides a fault detection service with higher reliability than the time-series SR, but with fault testing conducted only after each process run completes.

## Introduction

Integrated Circuit (IC) manufacturing has played an important role in the development of the Information Technology (IT) industry. In recent years, it has seen two major trends. Firstly, more and more transistors are being built per wafer [1]. Secondly, larger diameter wafers are being employed to increase the IC yield. Compared with current 300 mm diameter wafers, 450 mm diameter wafer technology is proposed as a main-stream product for the near future [2]. These developments require that control mechanisms in IC fabrication become more precise, year by year.

IC fabrication is a very complex process, with plasma etching as one of its fundamental process steps. The etching process impacts the quality of the final product output significantly and poses a range of research challenges. Four challenge types were mentioned in [3]: selectivity between etch mask and substrate, profile control of the etch pattern, damage to the material during etching and etch-rate control. Other important factors impacting the process were also identified, such as control of plasma chemistry, surface temperature, and pressure. As there remains a shortcoming in precise understanding of the underlining physical/chemical reactions involved, the process is often operated and controlled on empirical principles [4]. In order to monitor the process to effect its control, suitable process data collection mechanisms are required. The Optical Emission Spectrometer (OES) is a popular technology for this purpose. In the etching chamber, physical and chemical reactions trigger optical emissions. Different chemical species exhibit different spectrums. By observing the spectrum, etching progress can be inferred, in real-time. Compared with other measurement methods, OES provides non-intrusive measurements where no interference with the process is introduced. On the other hand, OES has limitations. High information complexity and redundancy of the data and difficulty in emission line identification are two well-known challenges [5]. Relating to these challenges, considerable OES-related research has been carried out including, virtual metrology methods [6], [7], endpoint detection strategies [8], [9] and system condition monitoring [10].

**Figure 1:**
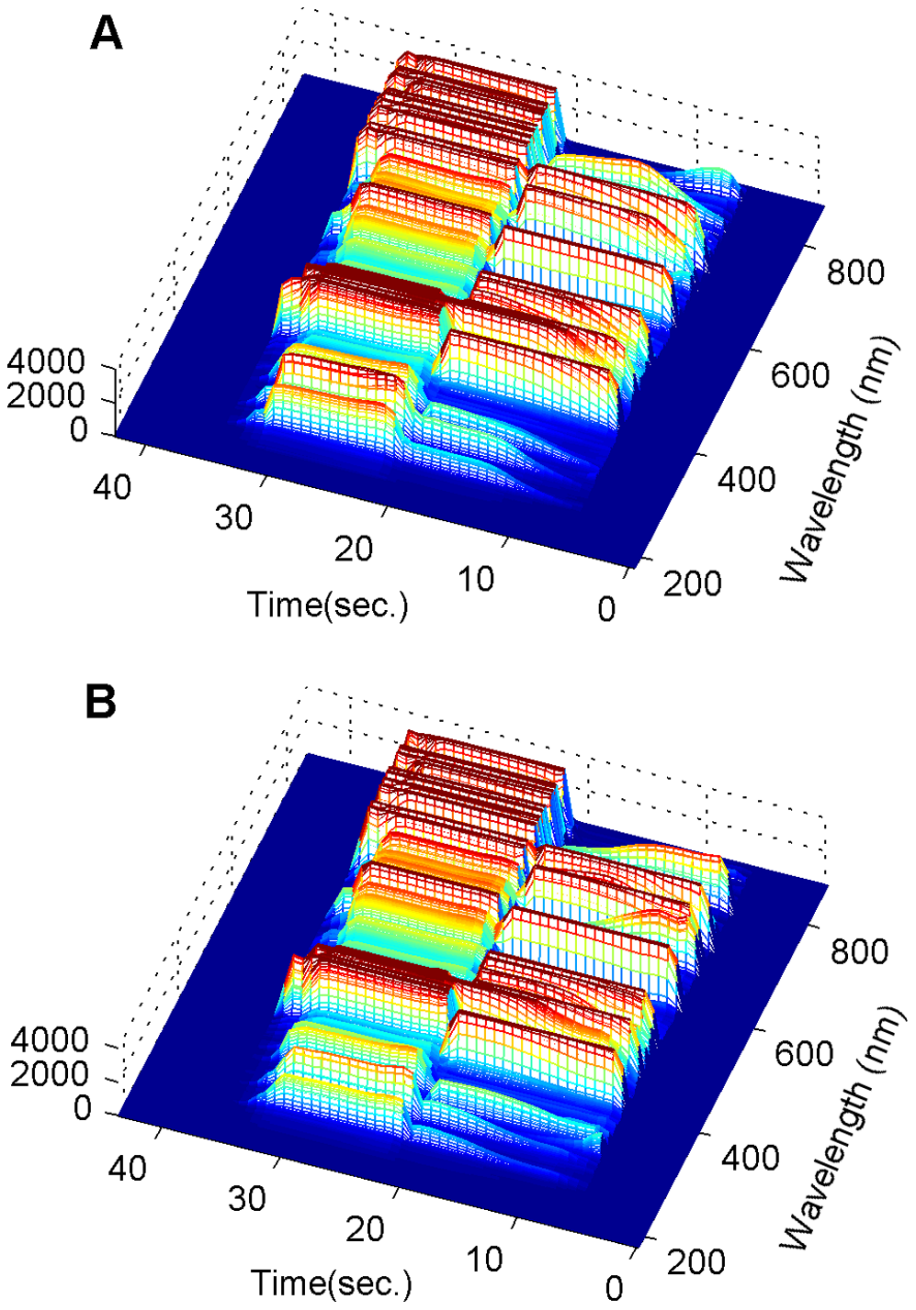
Comparison of time series OES between healthy sample and faulty sample. Panel A represents the time series OES measurements of a healthy etching process. Panel B represents the time series OES measurements of a faulty etching process. No significant difference can be easily observed.

This paper focuses on another important research topic in plasma etching, Fault Detection (FD). There are four major reasons for conducting fault detection in the IC fabrication process [11] : (1) improvement of process quality, (2) decrease of equipment downtime, (3) improvement of wafer quality and (4) less usage of testing wafers. Traditional FD technologies have two common problems: high cost and long-time delay before detection of a fault. For example, the Scanning Electron Microscopy (SEM) is used to measure etch depth, and then mean etch rate is calculated by the depth divided by the total etch time. This etch rate is a popular statistic to assess the process and wafer quality, however, this method introduces a large cost. The method also needs to wait for the end of the etching process, so a long-time delay is involved. The typical time delay to produce the etching result with traditional metrologies was demonstrated in [12], often taking hours or even days. During that time period, thousands of wafers can be damaged due to continuance of the same underlying system fault condition. Due to these problems, OES datasets have been widely studied for the purpose of fault detection due to two important features: its real-time monitoring capability and non-intrusive nature, however, the method poses its own challenges. As with other research with OES data, OES fault detection also suffers from the high dimensionality problem. To illustrate this, time series OES measurements from a healthy and a faulty etching process are presented respectively in Figure 1. It is difficult to tell the difference between them by a direct observation, so effective data processing methods are required to extract useful information from the raw data. In [13], the author mentioned that etch quality was affected significantly by small variations in equipment, and it is difficult to separate such a small variation from background noise for fault detection. In [14], process shift was discussed as another common challenge, for example, process shift can lead to a high rate of false alarms. (A typical scenario of this problem is presented and discussed in the result and discussion section, based on a real dataset). Due to these challenges, a large amount of research has focused on fault detection using OES, however, most approaches share the following common issues.

In general, most methodologies employ a fault detection test only after the whole etching process has completed. Starting with a dataset from a complete process run, popular FD methods make use of Principal Component Analysis (PCA) [15], support vector machines [16], pattern recognition methods [13], Independent Component Analysis (ICA) [17], and artificial neural networks [18]. Each of these methods can have their own particular problems. For example, two PCA issues were discussed in [11]. Firstly, the nonlinear characteristics of semiconductor plasma processes made the traditional PCA approach difficult to implement. Secondly, the PCA usually needs two control charts for the fault detection: Hotelling’s T^2^ and Squared Prediction Error (SPE). This can increase operation cost significantly, compared with a single chart. Regarding the use of ICA, it is always difficult to pick the component number and order in practice [17].

Some methodologies are able to detect faults at an early-stage with a single spectrum scan, but the times when the spectrums are taken are usually limited to a few chosen time points. In [19], a single OES scan was used for fault detection and classified at eight fixed time points. PCA was used in this case, but low FD accuracy was obtained. Therefore, the author concluded that a single OES scan could not provide a reliable FD service. In [12], a pattern recognition method was used for FD with OES. By calculating a matching rate between the testing sample and a library representing healthy samples, a fault could be detected successfully. Originally, the matching rate took all OES scans of a complete etching process as input, so the fault could only be detected at a post-process phase. This method could be adjusted to take individual scans as input, potentially. However, it still has the problem that the OES measurements were limited to certain time points. The authors built the library for healthy samples using average intensity plus/minus 3 standard deviations, did not discuss why 3 standard deviations was an appropriate choice.

In order to address these problems, a Similarity Ratio Analysis (SRA) method is proposed in this paper. Compared with previous research, the SRA method can detect faults at an early stage of the etching process. The OES data are also not limited to certain time points by the SRA, so the FD system can be more flexible because the timetable for the OES sampling can be adjusted based on real-time system feedback. The SRA method is based on a supervised training framework. In training, a range of acceptable wavelength intensities is calculated and stored based on healthy samples, called the SRA Library. The library describes features of an entire etching process versus time. In testing, an individual spectrum scan is compared with the library, and then a Similarity Ratio (SR) is calculated. The SR quantifies how similar the testing data and the library are, as a percentage score. System faults are detected by checking the SR values with certain conditions. A confidence level is also provided as a reference for the system alarm triggered. According to the result of the example dataset, this method can give an alarm for a system fault at about the 8.5 seconds in a 50 second faulty etching process.

## Method

The SRA model for fault detection is based on a supervised training framework. A training dataset is used to build a library for healthy samples. In the context of plasma etching, the sample is defined as a time series of OES spectrum scans for a complete etching process on one wafer. In real-time fault detection, a single testing spectrum scan is compared with the library at all time points. A corresponding SR value is calculated to quantify the similarity between them. Then a fault detection mechanism is applied based on the SR value, named 3-Warning-1-Alarm (3W1A). It provides a reliable fault reporting mechanism, by reducing the number of false alarms. The user is also given an opportunity to adjust the threshold value to trigger a warning and the number of warnings that trigger an alarm. The configuration can thus trade-off between the system sensitivity and the cost of a false alarm. In the rest of this section, calculation of the SRA library is firstly discussed, followed by a description of the SR calculation method. Then the FD mechanism is finally described.

### SRA Library Calculation

In the training phase of the SRA model, a SRA library is created to depict common features shared by all healthy samples. Each OES spectrum has intensity values at the same set of wavelengths, and a boundary function is computed for each wavelength individually. The boundary function describes the intensity range of healthy samples at each wavelength versus time. For each wavelength a pair of fitting functions describes the wavelength intensity upper boundary and lower boundary. The complete collection of functions of different wavelengths compose the SRA library, as expressed by Equation (1). The terms 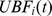 and 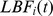 represent respectively the upper boundary function and the lower boundary function over time 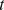 for the *i^th^* wavelength. The total number of wavelengths in the data is 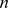.
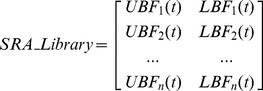
(1)


**Figure 2:**
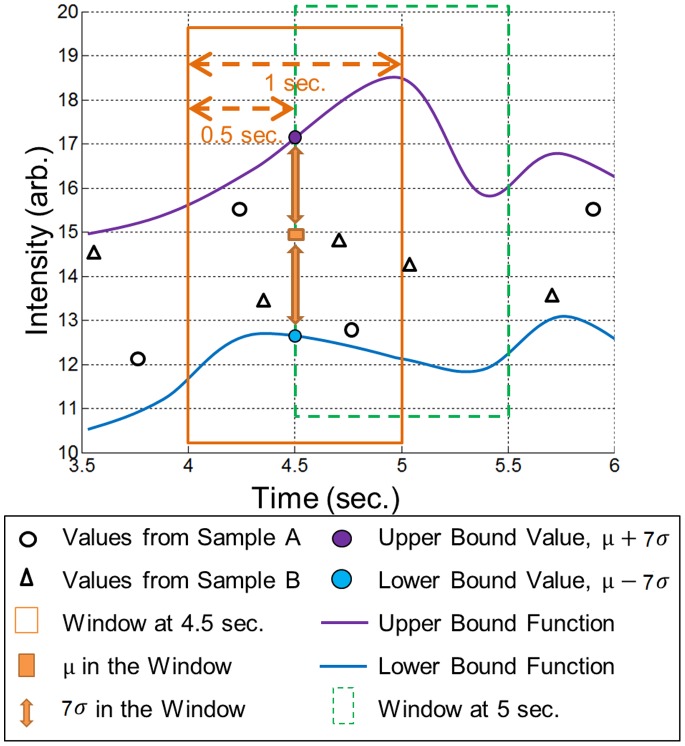
Illustration of boundary calculation for a single wavelength. Artificial data of two samples is demonstrated. A moving window is used to compute mean 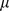 and standard deviation 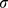 of intensity values inside the window. Upper and lower boundary values are calculated by 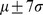. Corresponding fitting functions are calculated with the piecewise linear interpolation. The window size is set to 1.0 second and moves at a distance of half of its length, 0.5 seconds in this case.

The detailed calculation of the boundary functions for a single wavelength is illustrated in Figure 2. In our example dataset, each sample has a unique timetable to take spectrum scans, but all training samples derive from the same type of healthy etching process. Artificial data from only two samples is presented in the figure to provide a clear description of the method. Spectrums from different samples are re-ordered by time. A moving window is applied and average intensity 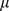 and standard deviation 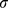, for each wavelength, are obtained within each window. Upper and lower boundary values are calculated at 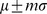, where 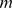 is determined empirically as a value of 7, which sets a suitable sensitivity for the fault detection algorithm (how 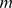 is determined from a given training dataset is discussed in the results section). Corresponding fitting functions are calculated from these distributed boundary values using a piecewise linear interpolation. The window size is set to 1.0 second. The window is moving at a distance of half of its length, 0.5 second in this case, such that the left half of every window overlaps with the previous window and the other half overlaps with the next window. According to experimental results, such a design gives a smooth fitting function. This fits the generic feature of plasma etching that wavelength intensity changes slowly throughout the whole process. Detailed discussion of the window size and sigma number is given in the section Results and Discussion. If spectrums scans from all samples are already well synchronised, the moving window is not needed. In that case, intensity values of the same wavelengths are grouped by the timestamps and corresponding boundary values are calculated in the same way.

### SR Calculation

The Similarity Ratio (SR) calculation is based on a comparison between the boundaries in the SRA library and individual spectrum scans. Upper bound values and lower bound values are calculated for each wavelength, using boundary functions in the library as described previously. 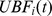 and 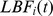 are respectively the upper bound value and lower bound value for the *i^th^* wavelength at time 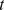. 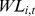 is the actual intensity value of this wavelength at time 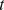 for the process being monitored. A similarity indicator 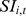 is used to record whether this wavelength at time 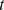
is between the boundaries, as per Equation (2). The same procedure is repeated for all wavelengths. Dividing by the total number of wavelengths 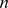, the 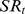 value at time 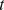 for a full spectrum scan is outputted as per Equation (3). The potential range of SR is from 0% to 100%. For example, an SR value of 90% means that 90% of the wavelengths of the sample spectrum are similar to the healthy library.
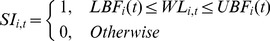
(2)

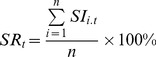
(3)


### Fault Detection Mechanism

**Figure 3:**
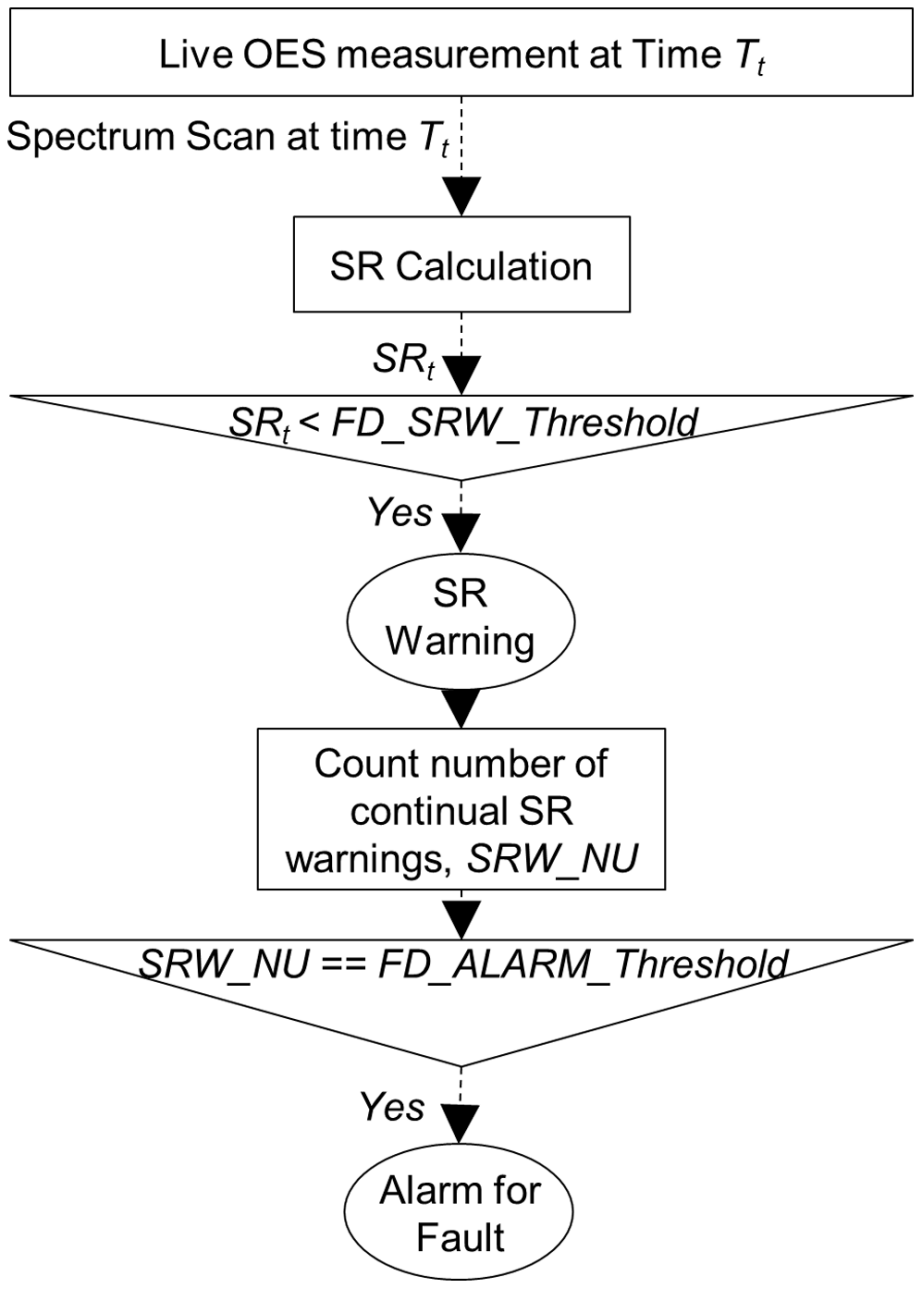
Workflow of the real-time FD with the SRA. A SR value is computed for each real-time spectrum scan. A SR warning is triggered if the SR value is below the SR Warning threshold (*FD_SRW_Threshold*). The warning is only registered internally, and it will not be sent to external process control applications. A high-level alarm can only be triggered, if there are a number of consecutive warnings. Consecutive number of warnings set as variable *FD_ALARM_Threshold*. The alarm will be sent to external applications, which would reply with necessary actions.

Considering the potential cost of false alarms (scrapping a healthy process run), a dedicated FD mechanism is used to provide a reliable and timely FD service. Plasma etching is a complex process with multiple physical and chemical reactions. Healthy etching processes cannot and do not need to be exactly identical to each other. Such tolerance is shown by a survey of a group of healthy samples in our data. These samples provide almost the same output quality, but can have quite different OES measurements. The SR measure is quite sensitive to such differences, so low SR values could be achieved even when the spectrum scan comes from a healthy process. If a system alarm is simply triggered by a single SR value below a threshold, there is a relatively high probability of a false alarm. In order to address this problem, multiple low consecutive SR values are used to trigger an alarm, using two control limits: an SR Warning threshold (*FD_SRW_Threshold*) and a consecutive warning count (*FD_ALARM_Threshold)*. A SR warning is triggered if the SR value is below the *FD_SRW_Threshold*. The warning is only registered in an internal log, and it will not be sent to external process control applications. A high-level alarm can only be triggered if the consecutive number of warnings exceeds the control limit *FD_ALARM_Threshold*. The alarm will be sent to external applications, which would reply with necessary actions, such as stopping the gas supply to the processing chamber. The detailed workflow of the method is presented in Figure 3 (A similar method was presented as Western Electric Rules [20]. Instead of using a single out-of-control data point, multiple data points were used in a control chart.). For the demonstration, the *FD_SRW_Threshold* is set to 90% and *FD_ALARM_Threshold* is set to 3. Based on this configuration, the FD rule is named 3-Warning-1-Alarm (3W1A) FD rule. Detailed experimental results for the 3W1A are presented in the next section. However, these two control limits can also be adjusted to customise the FD service with different sensitivities.

## Results and Discussion

The SRA model for fault detection is demonstrated using a real manufacturing dataset from an IC fabrication company. A 2-step etching process is monitored by an USB4000 Miniature Fibre Optic Spectrometer. A sample from the spectrometer is defined as a set of time-stamped spectrum scans for a complete etching process on one wafer. Every spectrum scan includes optical intensity measurements at 2048 different wavelengths from 178 nm to 874 nm. For each etching step, a different gaseous recipe is used. It takes approximately 25 seconds to finish one single step, so 50 seconds for the whole process in total. Gaseous species in the two steps include noble gases, fluorine and chlorine based compounds. There is a routine cleaning procedure between the two steps to remove residue from the first step. Reaction by-products can remain on the chamber wall, which is one of the major reasons for variance in the system output. An etch rate measurement is also included in every sample. Etch rate is a common metrology metric used to describe the output quality of an etching process but is difficult to measure, so it is impracticable to use it for real-time FD purposes. In the demonstration of the SRA model, it is only used as a reference for selection of healthy samples and faulty samples for evaluating the accuracy of the method. It is not needed after the SRA library is built. Actually, the SRA model does not need such a precise etch rate measurement for training. Any information which can identify whether a sample is good or bad is enough, such as knowledge based on some practical experiments of the process operators. This feature gives fewer constraints and more flexibility to the usage of the model.

**Figure 4:**
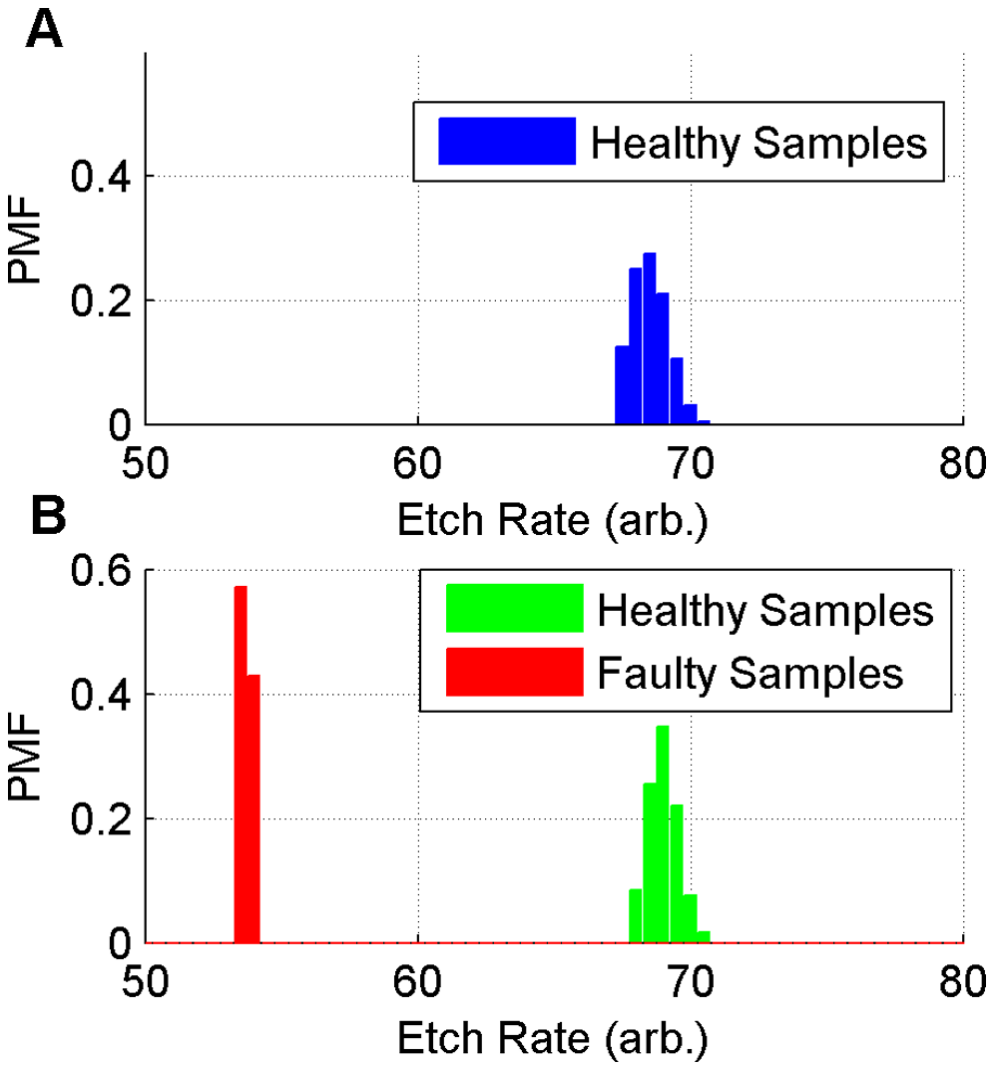
The PMF plots of etch rate values in training samples and testing samples. Panel A represents the PMF of training samples, which only include healthy samples. Panel B represents the PMF of testing samples, which include healthy samples, similar to the training samples, and faulty samples.

For model training, 200 healthy samples are selected. The SRA library is built on these samples for a healthy etching process. For model testing, another 118 healthy samples are used, as well as 7 faulty samples. These faulty samples are determined from detailed defect analysis of the product. All healthy samples share similar etch rates of around 69 arb. units. All faulty samples have etch rates of around 54 arb. units. The distribution of etch rates is presented in Figure 4 as Probability Mass Function (PMF) plots. Healthy samples from training and testing datasets have similar distributions, while the faulty samples are distinguished from the healthy ones. In the following model examination, this data selection helps to validate whether the model triggers a positive alarm for the faulty samples, and does not trigger false alarms for the healthy samples.

### SRA Result

#### Survey of process shift & moving window size selection

**Figure 5:**
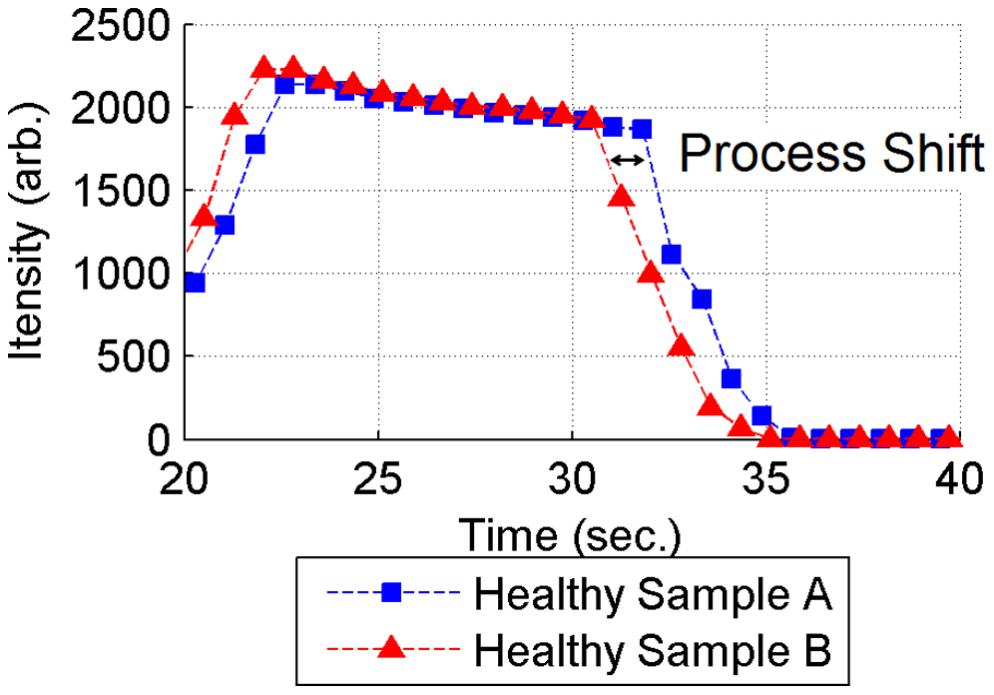
Illustration of process shift between two healthy samples with similar etch rates at wavelength 253.29 nm. Etch rates of both sample A and B are the same, 67.55 arb. unit, which implies that a similar etching output quality is shared by them. Shapes of the two curves are similar to each other, but there are differences in time series. A clear time shift can be observed.

Process shift is a very common practical problem in modelling of the plasma etching process. It is usually caused by small changes in a system variable [14]. It also can be caused by the sensor readings being taken at a different phase for different samples. It normally does not affect the overall properties of the output data, but it can lead to slightly different measurements at the same time point in different process samples. In Figure 5, process shift is illustrated at wavelength 253.29 nm for two samples from our data. These two samples have very similar etch rate values, about 67.548 arb. units for both of them. They also share very similar curve shapes, which imply similar etching features. But a shift can be observed between them versus time. A significant modelling bias could be introduced by the shift, if the model assumed synchronised measurement time points in all samples.

In the previous section on method description, a moving window is introduced to calculate boundary values based on the mean and standard deviation of the values falling within a window. This produces a smooth boundary function by taking major features of the data and removing abnormal outliers. This is consistent with an etching feature that the whole chemical environment is changing slowly across the whole process. Considering the process shift problem, the moving window can also include all shifted values whose shifting distances are less than the window size. So the outputted boundary functions are capable of resolving the shift problem. Theoretically, the window size should be large enough to include all values at the same process phase, but too large a window size will lead to a reduction in detail in the time series information.

**Figure 6:**
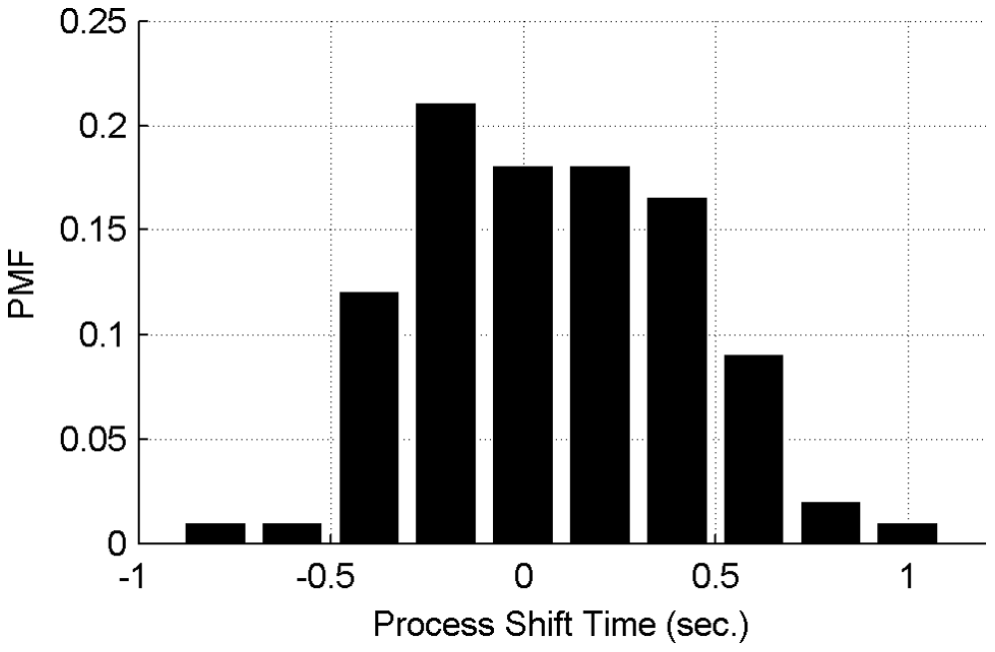
The PMF plot of process shift time in training samples. 10.55% of samples have shift distance less than 0.1 second, 51.26% of shift distances are less than 0.5 second, 86.43% of shift distances are less than 1.0 second, 96.48% of shift distances are less than 1.5 seconds, and 99.50% of shift distances are less than 2.0 seconds.

In order to find a suitable window size, a comprehensive survey is conducted for the relevant time shift of all training sample time series. One sample is selected out as a reference. The other samples are compared against the reference sample with a cross-correlation analysis, and the relevant shift time is calculated. Statistical results are presented in Figure 6. According to this plot and corresponding statistical results, 86.43% of process shifts concentrate on the range from about −0.5 second to 0.5 second. Hence, most shift distances in the example dataset would be covered by using a window size of 1.0 second. In that case boundary values will be computed every second. Other methods to solve this misalignment problem (e.g. Dynamic time warping, covariance optimized warping [21] and linear time scaling) normally need to wait for complete time series data and are computationally expensive [22], so they cannot be easily used for early-stage FD, unlike the proposed moving window method.

#### Sigma number selection

**Figure 7:**
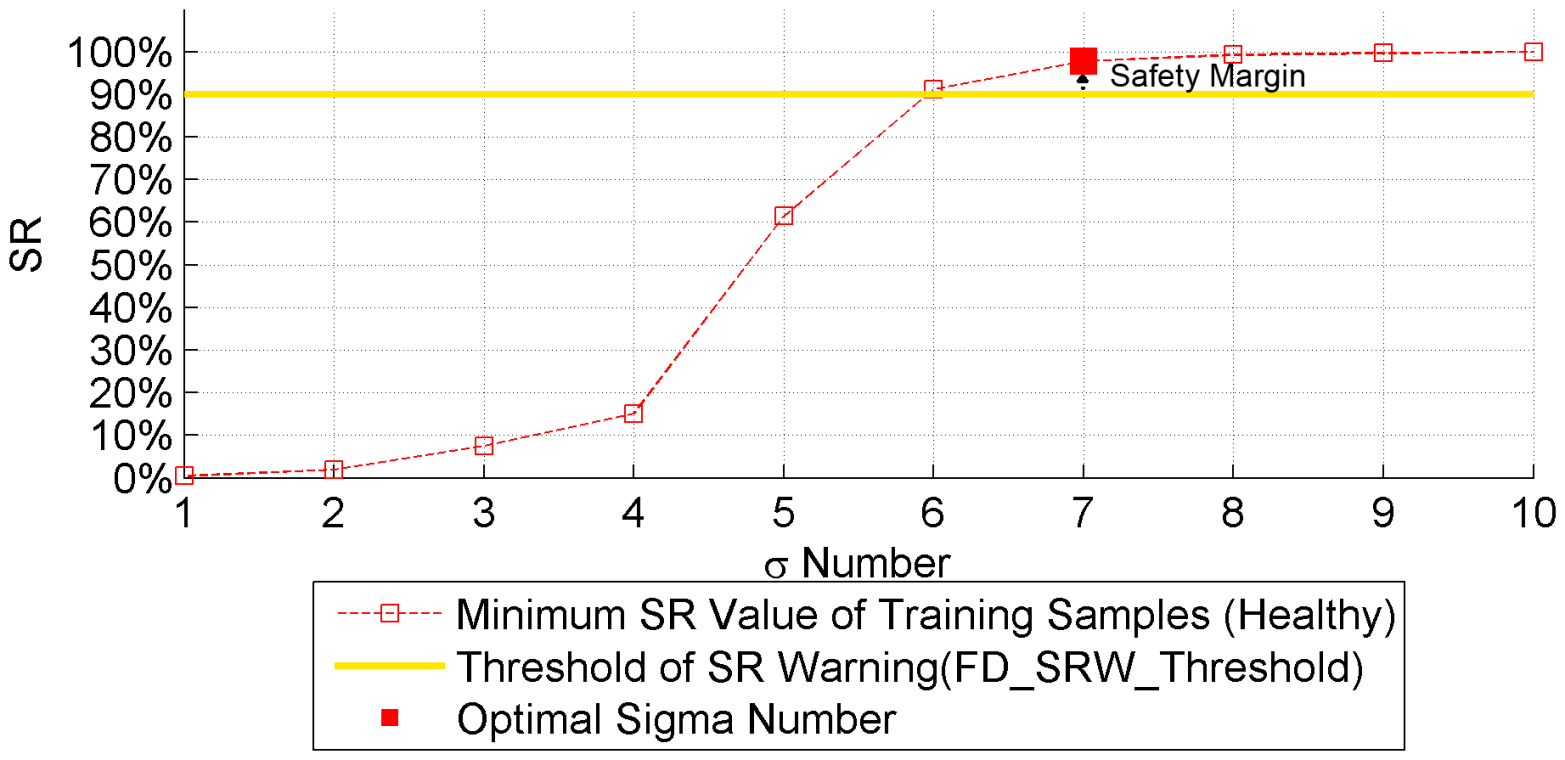
Plot of minimum SR values for training samples with different sigma numbers in the boundary function. By setting a selection threshold (warning threshold 90% plus a safety margin of 5%), sigma number 7 is selected as the smallest sigma number whose corresponding minimum SR value of training samples is higher than 95%.

The SRA library is built with 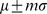 boundaries for each wavelength from healthy training samples. For each wavelength, 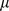 is the average value of all intensities falling in the moving window, and 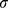 is the standard deviation. By choosing different sigma numbers 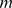, different SRA libraries are tested and the corresponding minimum SR values of training samples are presented in Figure 7. Because the SRA library is based on healthy training samples, the minimum SR values of these samples should be close to an SR value of 100% as far as possible. In that case the SRA library would more accurately represent healthy samples. On the other hand, the sigma number should not be too large as the library would then be too general to reliably distinguish between faulty and healthy samples. Hence, Figure 7 is used to decide a suitable sigma number based on selection of the warning threshold (*FD_SRW_Threshold*) for fault detection. This threshold is also used in the fault detection mechanim, as mentioned previously. In the following demonstration, the *FD_SRW_Threshold* is set to 90% plus a safety margin of 5% to increase the reliability of fault detection. The optimal sigma number is chosen to satisfy the following condition: it is the smallest sigma number whose minimum SR value is higher than *FD_SRW_Threshold* plus the safety margin. In that case, sigma number 7 is chosen with a minimum SR value of 99.32% in the training data set. A range of other threshold values have also been tested, and none triggers a false alarm. Multiple true alarms are triggered with most threshold values, except for thresholds below 20% or above 95%, due to either a too specific or too general SRA library.

### Fault Detection Result

Surveys of the window size and sigma number help to establish suitable choices for this particular dataset during the training phase: 1.0 second for the window size and 7 for the sigma number. In the testing phase, SR values are calculated for each spectrum scan of every healthy and faulty testing sample. For this particular dataset, a customised FD rule (3W1A) is applied to these SR values to provide a reliable fault detection service. In the 3W1A, a SR warning is triggered if a SR value is below the *FD_SRW_Threshold* which is equal to 90%. An alarm is triggered if three (*FD_ALARM_Threshold*) consecutive SR warnings occur. Control limits *FD_SRW_Threshold* and *FD_ALARM_Threshold* can also be customised based on the FD sensitivity required by users. For example, *FD_SRW_Threshold* can be increased to 99.99% and *FD_ALARM_Threshold* can be reduced to 1. So SR values below 99.99% will trigger a warning, and the warning will trigger an alarm immediately. This configuration is suitable when a very sensitive FD system is required and there is only a small cost for a false alarm.

In the following section, fault detection results with the 3W1A are discussed first for each individual spectrum scan, called time-series SR. Then average SR values for a complete etching process are presented, called post-process SR.

#### Time-series SR

**Figure 8:**
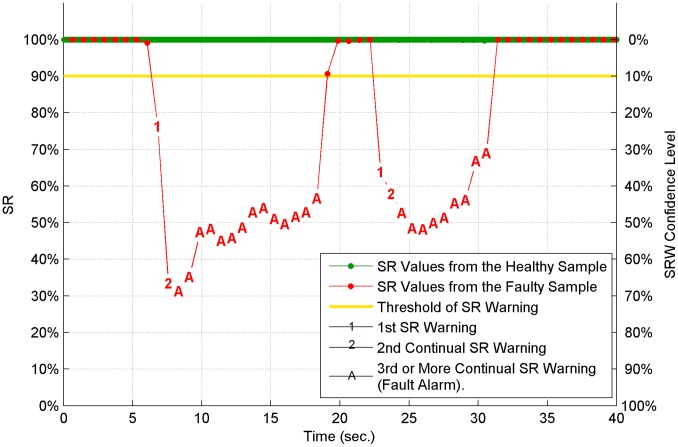
Fault detection result with the optimal sigma number 7. All 118 healthy testing samples and one typical faulty testing sample are presented. For the healthy samples, no alarm is triggered. All of them overlap with each other and have similar SR values which are close to SR value 100%. For the faulty sample, multiple faulty alarms are triggered from an early-stage in the process.

Fault detection results are demonstrated with all healthy testing samples and a typical faulty testing sample in Figure 8. The other faulty samples are quite similar to this one. Each sample includes a SR time series for a complete etching process. The SR values of the healthy sample are drawn in green, and the SR values of the faulty one are drawn in red. The SR warning threshold (*FD_SRW_Threshold*) is marked as a yellow straight line. All dots represent SR values which are above the threshold. Number 1 represents the first SR warning, whose value is below the threshold. The second consecutive SR warning is marked as number 2. The system alarm (the third consecutive SR warning) is marked as the letter A. For the healthy samples which overlap in the figure, no warning or alarm is triggered and all SR values are very close to SR value 100%. This high similarity between SRA library and healthy testing data implies that the SRA library is carrying enough features to describe the entire healthy etching process. For the faulty sample, multiple warnings and alarms can be observed. Beside the vertical SR axis on the left, an extra confidence level axis is also provided. It is calculated as 100% minus the SR value. It gives the users extra information about the alarms triggered. The lower the SR value is, the higher confidence the alarm is correct. For each faulty sample, the first warning occurs at about 7 seconds and the first alarm occurs at about 8.5 seconds for etching step 1. Considering all faulty samples, early-stage fault detection is achieved with 100% accuracy. No alarm is triggered at the beginning of etching step 1, transition from step 1 to step 2, or at the end of the step. This phenomenon is consistent with the etching feature that optical emission is weak at these three time periods, so no significant difference can be found between samples.

**Figure 9:**
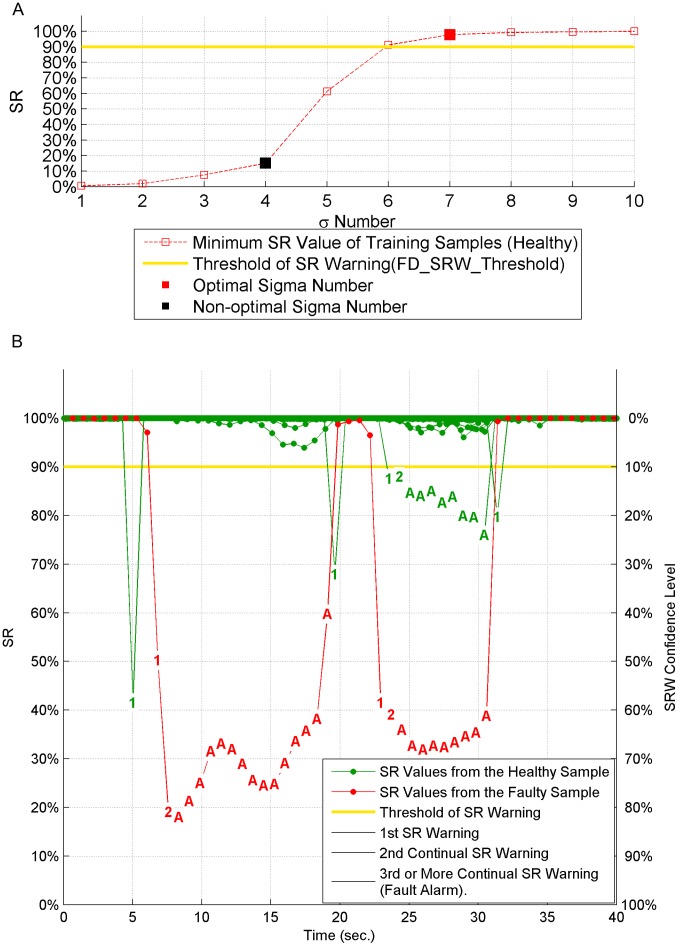
Fault detection result with non-optimal sigma number of 4. All 118 healthy testing samples and one typical faulty testing sample are presented. For the healthy samples, multiple warnings and alarms are triggered.

We note that sigma number selection is an important factor which impacts the fault detecion results significantly. Accurate fault detection results presented in Figure 8 validate that the method for sigma number selection (*the smallest sigma number whose minimum SR value should be higher than FD_SRW_Threshold plus a safety margin.*) is successful. If *FD_SRW_Threshold* is still set to 90% but sigma number 4 is chosen by mistake, for example, instead of 7, corresponding fault detection results are shown in Figure 9. The results show that incorrect sigma number selection leads to multiple false alarms (green letter A in plot) triggered by healthy samples.

#### Post-process SR

**Figure 10:**
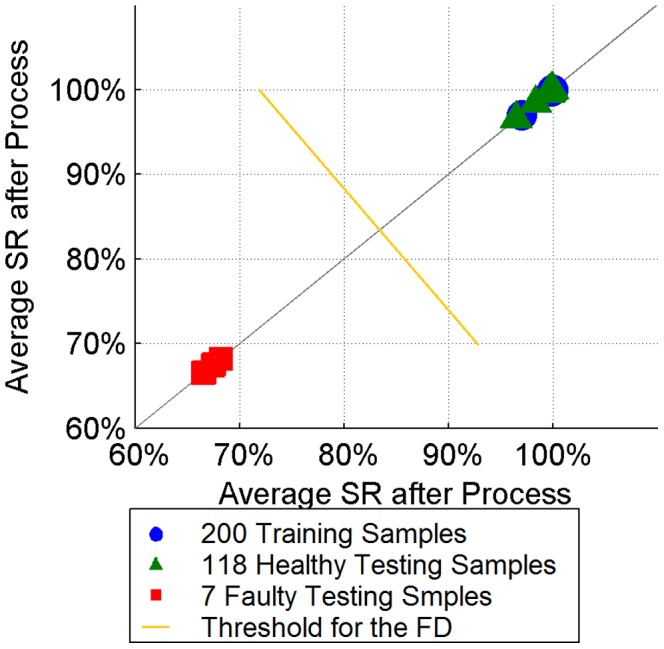
The average SR value plot of an entire etching process with training and testing dataset. All training samples and healthy testing samples have average SR values around 98%. All faulty testing samples have average SR values around 68%. Faulty samples can be easily identified from the healthy ones by using a simple threshold (yellow line) between them.

The average SR values for a complete etching process are also calculated based on the full SR time-series. The result is presented for training samples, healthy testing samples and faulty testing samples separately in Figure 10. All training samples and healthy testing samples have average SR values of around 98%. All faulty testing samples have average SR values of around 68%. Faulty samples can be easily identified from the healthy ones by using a simple threshold between them. Compared with the SR time-series, a fault reporting strategy like the 3W1A is not needed, and a bigger SR gap provides a more reliable fault detection result overall. An average SR may be a good option, when the early-stage fault detection is not required.

## Conclusions

We have shown that the proposed SRA method is effective for early-stage fault detection with a real manufacturing OES dataset from a plasma etching process. For these types of processes, early-stage detection can help to reduce overall process cost. In our method, spectrum scans are not limited to certain time points but use all available time-domain data, where process shift is accounted for by using a windowing method. Based on a trade-off between the potential cost caused by false alarms and time delay before correct alarms are raised, users can customise the sensitivity of the model.

Our future work will extend the SRA method. Firstly, fault classification can be realised based on the features of time-series SR from faulty samples (e.g. time when fault occurs, key wavelengths which significantly contribute to low SR values). This could help to quickly identify the underlying reasons causing the system failure. Sensitivity of the SR could also be improved, if wavelength numbers can be narrowed down for a certain fault type. Secondly, the SRA method could be combined with dimension reduction methods to reduce the input data size, which could accelerate system response times. Thirdly, SRA will be further developed and tested with additional system monitoring variables besides OES data, such as temperature in the processing chamber.

## References

[1] Moore GE (1998) Cramming more components onto integrated circuits. Proceedings of the IEEE 86: 82–85.

[2] International Technology Roadmap for Semiconductors. Available: http://www.itrs.net/about.html. Accessed 2013 Mar 2.

[3] Cardinaud C, Peignon MC, Tessier PY (2000) Plasma etching: principles, mechanisms, application to micro-and nano-technologies. Applied Surface Science 164: 72–83.

[4] Edgar TF, Butler SW, Campbell WJ, Pfeiffer C, Bode C, et al (2000) Automatic control in microelectronics manufacturing: Practices, challenges, and possibilities. Automatica 36: 1567–1603.

[5] Mackus A, Heil S, Langereis E, Knoops H, Van de Sanden M, et al (2010) Optical emission spectroscopy as a tool for studying, optimizing, and monitoring plasma-assisted atomic layer deposition processes. Journal of Vacuum Science & Technology A: Vacuum, Surfaces, and Films 28: 77–87.

[6] Zeng D, Spanos CJ (2009) Virtual metrology modeling for plasma etch operations. Semiconductor Manufacturing IEEE Transactions on 22: 419–431.

[7] Lynn S, Ringwood J, Ragnoli E, McLoone S, MacGearailty N (2009) Virtual metrology for plasma etch using tool variables. Advanced Semiconductor Manufacturing Conference IEEE: 143–148.

[8] Yue HH, Qin SJ, Wiseman J, Toprac A (2001) Plasma etching endpoint detection using multiple wavelengths for small open-area wafers. Journal of Vacuum Science & Technology A: Vacuum, Surfaces, and Films 19: 66–75.

[9] Westerman R, Johnson D, Lai S, Teixeira M (2006) Endpoint detection methods for time division multiplex etch processes. MOEMS-MEMS 2006 Micro and Nanofabrication: 61090I–61090I.

[10] Bacelli G, Ringwood JV (2007) Tracking plasma etch process variations using Principal Component Analysis of OES data. 4th International Conference on Informaticas in Control, Automation and Robotics. In press.

[11] Yu J (2011) Fault detection using principal components-based Gaussian mixture model for semiconductor manufacturing processes. Semiconductor Manufacturing IEEE Transactions on 24: 432–444.

[12] Yang R, Chen R (2010) Real-time plasma process condition sensing and abnormal process detection. Sensors 10: 5703–5723. 22219683 10.3390/s100605703 PMC3247728

[13] Chen MS, Yen T, Coonan B (2004) Real-time fault detection and classification for manufacturing etch tools. Semiconductor Manufacturing Technology Workshop Proceedings IEEE: 103–106.

[14] Yue HH, Tomoyasu M (2004) Weighted principal component analysis and its applications to improve FDC performance. Decision and Control IEEE: 4262–4267.

[15] Wise BM, Gallagher NB, Butler SW, White DD, Barna GG (1999) A comparison of principal component analysis, multiway principal component analysis, trilinear decomposition and parallel factor analysis for fault detection in a semiconductor etch process. Journal of Chemometrics 13: 379–396.

[16] Sarmiento T, Hong SJ, May GS (2005) Fault detection in reactive ion etching systems using one-class support vector machines. Advanced Semiconductor Manufacturing Conference and Workshop IEEE: 139–142.

[17] Lee JM, Qin SJ, Lee IB (2006) Fault detection and diagnosis based on modified independent component analysis. AIChE Journal 52: 3501–3514.

[18] Hong SJ, May GS (2005) Neural-network-based sensor fusion of optical emission and mass spectroscopy data for real-time fault detection in reactive ion etching. Industrial Electronics, IEEE Transactions on 52: 1063–1072.

[19] Yue HH, Qin SJ, Markle RJ, Nauert C, Gatto M (2000) Fault detection of plasma etchers using optical emission spectra. Semiconductor Manufacturing IEEE Transactions on 13: 374–385.

[20] Western Electric Company Inc. (1958) Statistical quality control handbook. Western Electric Company Inc. 24–28 p.

[21] Tomasi G, van den Berg F, Andersson C (2004) Correlation optimized warping and dynamic time warping as preprocessing methods for chromatographic data. Journal of Chemometrics 18(5): 231–241.

[22] Zhang Y, Lu B, Edgar TF (2013) Batch trajectory synchronization with robust derivative dynamic time warping. Industrial & Engineering Chemistry Research. 52(35): 12319–12328.

